# CD24^+^ cells fuel rapid tumor growth and display high metastatic capacity

**DOI:** 10.1186/s13058-015-0589-9

**Published:** 2015-06-04

**Authors:** Ran Rostoker, Sagi Abelson, Inna Genkin, Sarit Ben-Shmuel, Ravi Sachidanandam, Eyal J. Scheinman, Keren Bitton-Worms, Zila Shen Orr, Avishay Caspi, Maty Tzukerman, Derek LeRoith

**Affiliations:** Diabetes and Metabolism Clinical Research Center of Excellence, Clinical Research Institute at Rambam (CRIR) and the Faculty of Medicine, Technion, Rambam Medical Center, P.O.B 9602, Haifa, 31096 Israel; Laboratory of Molecular Medicine, Rambam Health Care Campus and Rappaport Faculty of Medicine and Research Institute, Technion, Haifa, 31096 Israel; Department of Oncological Science, Icahn School of Medicine at Mt Sinai and the James J Peters VA Medical Center, New York, USA; Division of Endocrinology, Diabetes and Bone Disease, Icahn School of Medicine at Mount Sinai, New York, NY USA

## Abstract

**Introduction:**

Breast tumors are comprised of distinct cancer cell populations which differ in their tumorigenic and metastatic capacity. Characterization of cell surface markers enables investigators to distinguish between cancer stem cells and their counterparts. CD24 is a well-known cell surface marker for mammary epithelial cells isolation, recently it was suggested as a potential prognostic marker in a wide variety of malignancies. Here, we demonstrate that CD24^+^ cells create intra-tumor heterogeneity, and display highly metastatic properties.

**Methods:**

The mammary carcinoma Mvt1 cells were sorted into CD24^−^ and CD24^+^ cells. Both subsets were morphologically and phenotypically characterized, and tumorigenic capacity was assessed via orthotopic inoculation of each subset into the mammary fat pad of wild-type and MKR mice. The metastatic capacity of each subset was determined with the tail vein metastasis assay. The role of CD24 in tumorigenesis was further examined with shRNA technology. GFP-labeled cells were monitored in vivo for differentiation. The genetic profile of each subset was analyzed using RNA sequencing.

**Results:**

CD24^+^ cells displayed a more spindle-like cytoplasm. The cells formed mammospheres in high efficiency and CD24^+^ tumors displayed rapid growth in both WT and MKR mice, and were more metastatic than CD24- cells. Interestingly, CD24-KD in CD24+ cells had no effect both in vitro and in vivo on the various parameters studied. Moreover, CD24^+^ cells gave rise in vivo to the CD24^−^ that comprised the bulk of the tumor. RNA-seq analysis revealed enrichment of genes and pathways of the extracellular matrix in the CD24^+^ cells.

**Conclusion:**

CD24^+^ cells account for heterogeneity in mammary tumors. CD24 expression at early stages of the cancer process is an indication of a highly invasive tumor. However, CD24 is not a suitable therapeutic target; instead we suggest here new potential targets accounting for early differentiated cancer cells tumorigenic capacity.

**Electronic supplementary material:**

The online version of this article (doi:10.1186/s13058-015-0589-9) contains supplementary material, which is available to authorized users.

## Introduction

Breast tumors frequently comprise heterogeneous cancer cells with distinct morphologic and phenotypic features [1, 2]. Intra-tumor heterogeneity can arise from stochastic genetic or epigenetic changes, or can be attributed to signals from the stroma within the tumor [3, 4]. More recently, the cancer stem-cell hypothesis was proposed to explain these cancer cells heterogeneity and hierarchical organization [5, 6]. From a clinical perspective, targeting specific cell lineage with metastatic proclivity remains a life-saving therapeutic challenge, as most breast tumors are invasive and result in a poor prognosis with decreased disease-free survival.

The variable expression of cell surface markers among cancer cells is being widely exploited to identify, isolate and characterize distinct cancer cell populations [7, 8]. CD24, an anchored cell surface glycoprotein was recently identified as an ideal marker to isolate pure mammary epithelial cells that can be further isolated, along with staining for other cell surface markers, into stem/progenitor cells. In line with that finding, isolated Lin^−^CD24^+^CD49f murine mammary cells have been shown capable of generating functional mammary tissue in vivo [9, 10]. As a ligand of p-selectin, CD24 serves as an adhesion molecule that facilitates the metastatic process by supporting the rolling of cancer cells on activated platelets and endothelial cells [11, 12]. Recently it was suggested that although CD24 lacks an intracellular domain, it is involved in regulating cancer cell proliferation and gene expression. However the mechanisms mediating these effects remain elusive [13].

Based on CD24 expression, we have recently identified two distinct subpopulations in the mammary carcinoma Mvt-1 cell line, which is derived from a primary mammary tumor in MMTV-VEGF/c-myc bi-transgenic female mice. Although several studies suggest that it is the lack of CD24 expression that characterizes breast cancer stem cells [14, 15], it is known that cell-surface markers are not conserved among different tumors, due to differences in the driver mutations [4]. Several questions remain to be on the role of CD24 in cancer and more specifically in tumor heterogeneity. First, does CD24 actively mediate tumorigenesis, or does it serve only as a surface marker for tumorigenic cells? Answering this would facilitate the design of better therapeutic strategies, i.e., inhibition/downregulation of CD24 or alternatively exploiting its expression for targeting specific cancer cells. Second, do CD24^+^ cells act as stem/progenitor cells and are CD24^−^ cancer cells their progeny? Finally, are there specific genes that will discriminate between CD24^−^ and CD24^+^ cells, and are there changes at the protein level in these subpopulations such as phosphorylation that result in activation of different signaling pathways?

To begin to elucidate the cellular differences between distinct cancer cell subpopulations, we isolated two cancer cell subpopulations based on CD24 expression and phenotypically characterized these cell subsets. Next, we turned to mouse models to determine the tumorigenic capacity of each subset. To investigate the role of CD24 in mediating tumorigenesis, we knocked down CD24 expression with an shRNA construct. In addition, we demonstrated a degree of hierarchy and plasticity in these cancer cells. We further analyzed the gene expression profile of each cell subset and tested the implication of these findings in vivo.

Our results suggest that CD24 cell surface expression on mammary epithelial cancer cells identify a subpopulation of cells that is enriched with stem/progenitor-cell properties. These CD24^+^ cells display highly tumorigenic properties with high metastatic capacity; moreover, these cells can differentiate in vivo, hence creating intra-tumor heterogeneity. CD24^+^ cells differ from their counterpart in their gene profile and are characterized by elevated extracellular matrix gene expression; this may enable them to modify the soil of the host tissue in order to invade and form metastasis.

## Methods

### Cell culture

The mouse mammary cancer cell line, Mvt-1, has been previously described [16]. Cells were cultured in DMEM (Biological Industries, Beit Haemek, Israel) supplemented with 10 % fetal bovine serum (Biological Industries) and antibiotics (penicillin, streptomycin; Biological Industries) at 37 °C in a humidified atmosphere consisting of 5 % CO2 and 95 % air.

### Animals

Female MKR mice and control mice on an FVB/N background were used in this study. The MKR mice are transgenic with a dominant-negative insulin-like growth factor-I receptor specifically targeted to the skeletal muscle with a resultant severe insulin resistance and hyperinsulinemia phenotype [17]. Mice were kept on a 12-hour light/dark cycle with access to standard mouse chow and fresh water ad libitum. Mice studies were performed according to the protocol approved by the Technion Animal Inspection Committee. The Technion holds an National Institutes of Health (NIH) animal approval license number A5026-01.

### Flow cytometry

The following antibodies were used for cell surface staining of the Mvt-1 cell line, Pacific-Blue-conjugated anti-CD24, PE-conjugated anti-CD29, AF647 (Alexa Fluor 647)-conjugated anti-CD61/β3 and AF647 (Alexa Fluor 647)-conjugated anti-CD49F (Biolegend, San Diego, CA, USA): 7-Amino actinomycin D (7-AAD, Biolegend) was used to gate live cells. Cells were stained at a concentration of 5 × 10^6^ cells/ml of FACS buffer (PBS containing 0.1 % BSA) for 20 minutes on ice in the dark, after which the cells were washed twice and resuspended in FACS buffer containing 7-AAD. Stained cells were analyzed using the CyAn ADP Instrument (Dako-Cytomation, Glostrup, Denmark) and the FlowJo 7.25 analysis software. Intracellular staining was performed with the CytoWx/Cytoperm kit (BD PharMingen, San Diego, CA, USA), after CD24 cell surface staining. Cells were then washed with the Cytofix solution, and permeabilized with perm wash for 10 minutes on ice. To detect intracellular CD24, the antibody was added in combination with perm wash for 15 minutes on ice. Cells were washed twice and resuspended in FACS buffer until analysis. Flow cytometry-based cell sorting for CD24^−^ and CD24^+^ cells was performed using FACSAria (BD Biosciences, San Jose, CA, USA).

### Tumorspheres

CD24^−^ and CD24^+^ single cell suspensions were prepared and plated in nonadherent conditions at 600 cells/cm^2^ in DMEM F12 HAM medium (Sigma, Rehovot, Israel) containing 20 ng/ml bFGF (Sigma), 20 ng/ml EGF (Sigma), 4 μg/ml of Heparin (Sigma) and B-27 supplement (1:50 dilution, GIBCO, Burlington, ON, USA), and cultured at 37 °C with 5 % CO2. Tumorsphere-forming efficiency (percentage) was calculated after 5 days as follows: (number of tumorspheres (>50 mm in diameter) per well/number of cells seeded per well)*100. To assess self-renewal, primary tumorspheres were centrifuged at 115 × g for 5 minutes, the pellet was resuspended in 300 μl of 0.5 % trypsin/0.2 % EDTA for 3 minutes at 37 °C. Tumorspheres were disaggregated into single cell suspension with the use of a 25-G needle and syringe, (trypsin was neutralized with medium containing serum). Cells were centrifuged at 580 × g for 5 minutes, the pellet was resuspended in ice-cold PBS, and single cell suspension was assured under a microscope. Single cells were plated at the same seeding density that was used in the primary generation. Tumorspheres (>50 mm in diameter) were measured after 5 days in culture. Self-renewal was calculated by dividing the number of secondary tumorspheres formed by the number of primary tumorspheres formed.

### Quantitative PCR reaction for cDNA products

Quantitative PCR was performed using Absolute Blue SYBR-Green ROX mix (Thermo scientific, ABgene, Epsom, UK). RNA was extracted from treated Mvt-1 cells with the Total RNA Purification Kit (NORGEN Biotek Corp, Thorold, Canada) according to manufacturer’s instructions, followed by single-stranded cDNA synthesis using the Verso™ reverse transcriptase (Thermo Scientific, ABgene). The expression measurement of the designated genes was performed with the Rotor-GeneTM 6000 system (Corbett Research, Sydney, Australia) and its software, ver. 1.7. The relative gene copy number was normalized using *B2M* as the independent internal control gene, and calculated by the 2^-(Ct_(n)_-Ct_(normalizer)_) method, where Ct represents cycle threshold.

### Proliferation

Cells were seeded in 96-well plates (1,000 cells/well) for 48 h. Proliferation of the cells was quantitated by the CyQuant (Invitrogen, Carlsbad, CA, USA) fluorimetric DNA assay according to the manufacturer’s recommendations.

### Knockdown of CD24 by retroviral-based delivery of shRNA

The shRNA targeting sequence against mouse CD24, sense (5′-CCCAAATCCAAGTAACGCTACCATTCAAGAGATGGTAGCGTTACTTGGATTTGTTTTTA-3′) and antisense (5′-GGGTTTAGGTTCAT TGCGATG GTAAGT TCTCTACCATCGCAATGAACCTAAACAAAAAT-3′) or scrambled (control) oligonucleotides were annealed and then ligated into the BglII and HindIII sites of the psuper.retro.puro vector (OligoEngine, Seattle, WA, USA). Retroviral particles were generated and introduced as described above.

### Syngeneic orthotopic tumor models

CD24^−^ and CD24^+^ cells or knockdown cells were suspended in 100 μl PBS and then injected (5 × 10^4^ cells/mouse; fewer cells were injected for the serial dilution experiments) into the left inguinal mammary fat pad (number 4) of 8-week-old female MKR mice. Tumor volume was monitored once a week with calipers; volume was calculated in mm^3^ by the formula: (width2 × length × 0.5). Following sacrifice, tumors were removed and weighed, then flash frozen in liquid nitrogen and kept at −80 °C for further analysis.

### Generation of the green fluorescent protein-expressing (GFP) cell line

A construct containing GFP (NV-SV-40-puro-linkek-Ins-PGK-eGFP, a generous gift from Dr Neufeld, Technion, Haifa, Israel) sequence was transfected into the Lentiviral packaging cell line 293FT together with ViraPower packaging mix (Invitrogen) using the Lipofectamine 2000 reagent (Invitrogen). At 48 h post-transfection, the supernatant containing the viruses was collected and filtered through a 0.45-Am syringe filter. Viruses were used to infect Mvt1 cells in the presence of polybrene (Sigma, St Louis, MO, USA) at a final concentration of 8 μg/ml. Infected cells were selected and maintained with 2 μg/ml puromycin. CD24^−^/GFP^+^ and CD24^+^/GFP^+^ cells were sorted using FACSAria (BD Biosciences).

### Tumor dissociation into single cells

Breast tumors were minced with scalpels and transferred to gentleMACS™ dissociator C-tubes (Miltenyi Biotec, Bergisch Gladbach, Germany) containing 5 ml of DMEM (Biological Industries, Beit Haemek, Israel) supplemented with 10 % FBS (Biological Industries, Beit Haemek, Israel). C-tubes were then connected to the gentleMACS™ dissociator and tumor dissociation was performed according to the manufacturer’s instructions. Minced tumors were incubated in the C-tubes for 45 minutes with 300 units/ml collagenase I (Sigma-Aldrich, Rehovot, Israel) and 2 mg/ml dispase II (Roche Diagnostics, Mannheim, Germany) at 37 °C in a humidified atmosphere consisting of 5 % CO2 and 95 % air. Following incubation a second spin on the gentleMACS™ dissociator was performed, and the cells were then filtered through a 40-μm falcon strainer (Becton Dickinson, Franklin Lakes, NJ, USA).

### mRNA-seq

Total RNA was isolated and purified from cells using the Total RNA Purification Kit (NORGEN Biotek Corp) according to the manufacturer’s instructions. cDNA libraries were prepared using 1 μg of total RNA using the TruSeq RNA Sample Preparation Kit v2 (Illumina). Briefly, polyadenylated RNA was purified using magnetic beads and fragmented according to the manufacturer’s instructions. After ligation of the paired-end adapter the approximately 200-bp fraction was amplified with 15 cycles of PCR. cDNA libraries were subjected to the Illumina HiSeq 2500 platform to 50-bp single reads sequencing.

### Analysis of mRNA-Seq data

Custom-built software was used to map the reads to the mouse genome (mm9) and estimate the coverage of each gene. Briefly, the reads were clipped at both ends (2 at the 5′ end and 1 at the 3′ end) to remove potentially error-prone sites. The reads were then mapped to the genome using a suffix-array-based approach. The median of coverage across the transcripts was used as an estimate of gene expression. The expression values were quantile normalized, and ratios were calculated by comparing the mean of samples from CD24^−^ cells against the mean of samples from CD24^+^ cells. The noise (or limits) of expression detection in the mRNA-seq data is the peak of the distribution of expression values (genes with high expression are in the long tail of this distribution). The expression values were regularized by adding the noise to the expression of each gene before the ratios were calculated. This ensures that genes with low expression do not contribute to the list of genes with large fold-changes. Genes expressed differentially between the groups were selected by using a *p* value <0.05 (calculated by the *t* test) and fold-change of at least 2 was required for the gene to be included in the list. The heat map was generated by custom software R.

### Pathway analysis

We used Gene Ontology (GO) analysis [18] to analyze and compare enriched pathways in CD24^−^ and CD24^+^ cells. The Gorilla tool was used in order to identify enriched GO terms. As target list inputs, we used all the annotated genes as the background for the analysis. GO terms were selected with a conservative threshold of false detection rate (FDR) <0.2 and a *p* value <0.05.

### Tail vein metastasis assay

We injected10,000 cells from each subset through the tail vein of wild-type (WT) mice to assess lung metastatic activity. Mice were killed 28 days after injection, lungs were removed and fixed, and macrometastases were counted under a light-microscope.

### Statistical analysis

All data are presented as mean ± standard error of the mean (SEM). The independent *t* test and the Mann-Whitney test was used for statistical analysis of unmatched groups; the Wilcoxon signed-rank test was used for matched group comparison, with *p* values <0.05 considered statistically significant.

## Results

### Differential expression of CD24 observed within the Mvt1 cells

**Fig. 1:**
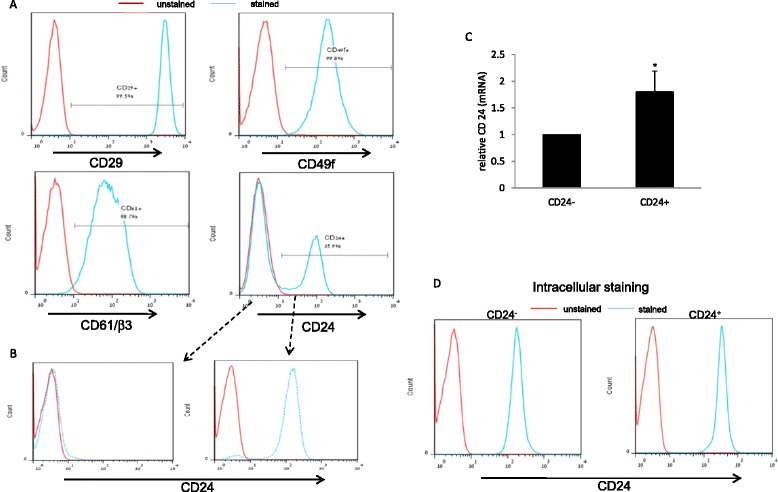
Isolation and characterization of two Mvt1 subgroups distinguished by their CD24 expression. **a** Fluorescence-activated cell sorting (FACS) showing CD29, CD49f, CD61/β3 and CD24 expression in the Mvt1 cell line. **b** Flow cytometric separation of Mvt1 cells based on CD24 expression. **c** RNA was extracted from the sorted CD24^−^ and CD24^+^ populations for CD24 quantitative RT-PCR analysis. **d** FACS showing CD24 intracellular expression. The Mann-Whitney test was performed to compare CD24^−^ and CD24^+^ cells. *p <0.05

In order to find heterogeneity and hierarchal organization in the mammary carcinoma Mvt1 cell line, we characterized the cell surface expression of CD29, CD61/β3, CD24 and CD49f, common markers for isolation of mammary cancer stem cells. Using flow cytometry analysis, we found uniform expression of CD29, CD61/β3 and CD49f in the Mvt1 cell line, however, CD24 analysis revealed two distinct populations, CD24^−^ cells (64.1 %) and CD24^+^ cells (35.9 %) (Fig. 1a). Next, we double-sorted the cells into pure (>95 %, as determined by FACS analysis (Fig. 1b)) CD24^−^ and CD24^+^ cells. Grown in complete media, CD24^+^ cells were widely dispersed in culture, displaying spindle-like cytoplasm, whereas CD24^−^ displayed a more rounded epithelial appearance (data not shown). It is important to note that these differences between CD24^+^ and CD24^−^ cells remained constant for more than 18 passages, whereas a dynamic shift was found in the Mvt1 cell line as a result of different growth rates of the two subpopulations. Next, we sought to determine CD24 expression at the transcript level by quantitative (q)RT-PCR in these two subpopulations. Indeed, CD24 mRNA levels were moderate but significantly increased in the CD24^+^ cells approximately 1.8-fold compared to the CD24^−^ cells (Fig. 1c). These results suggest that CD24 is expressed in both subpopulations, while cell surface expression differs significantly. Indeed, CD24 was expressed intracellularly in both CD24^−^ and CD24^+^ subpopulations (Fig. 1d).

### CD24^+^ cells display in vivo aggressiveness in both WT and MKR mice

**Fig. 2:**
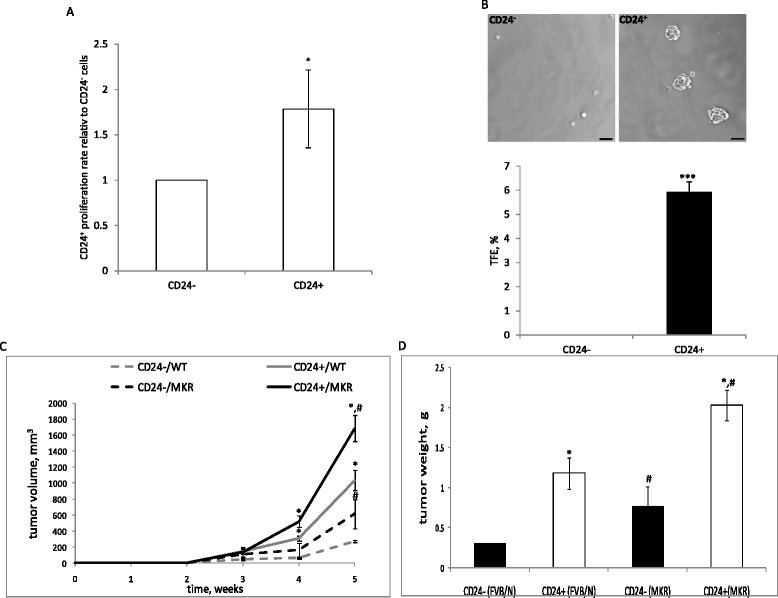
CD24^+^ cells display in vivo aggressiveness in both wild-type (*WT*) and MKR mice. **a** 1*10^3^ CD24^−^ and CD24^+^ cells were seeded and grown in full medium for 2 days, and relative proliferation rate between the cells was determined using the Cyquant proliferation assay. **b** Representative photomicrographs of a tumorsphere (>50 um diameter) grown from CD24^−^ and CD24^+^-single cells in non-adherent culture for 5 days (*upper pane*l). Percentage tumorsphere formation efficiency (*TFE %*) comparison between CD24^−^ and CD24^+^ (*lower panel*). The Mann-Whitney test was performed to compare CD24^−^ and CD24^+^ cells (*p <0.05). **c** CD24^−^ and CD24^+^ cells were injected into the fourth mammary fat pad of 8-week-old virgin *WT* and MKR mice (50,000 cells/mouse), and tumor volume was measured during a 5-week period. **d** Tumor weights were measured at necropsy. The Mann-Whitney test was performed to compare CD24^−^ and CD24^+^ tumors (*p <0.05, and to compare tumors in *WT* and MKR mice (^#^ *p* <0.05)

In order to further characterize the CD24^−^ and CD24^+^ cells, the CyQUANT cell proliferation assay was applied to compare the proliferation rates of CD24^−^ and CD24^+^ cells. We observed a significantly enhanced proliferation rate (>1.7-fold) for CD24^+^ cells compared to CD24^−^ cells (Fig. 2a). The tumorsphere assay correlates with tumor initiating cell ability and can be modified to assess self-renewal. We used this method to test CD24^−^ and CD24^+^ ability to form in vitro tumorspheres. Cells were cultured in non-adherent plates for 5 days in serum-free optimized medium. CD24^+^ cells formed relatively high numbers of tumorspheres, which were counted under a light microscope, and the percentage tumorsphere formation efficiency (TFE) was calculated (Fig. 2b). Primary tumorspheres generated by the CD24^+^ cells were disaggregated into single-cell suspension; these cells were able to form secondary tumorspheres, which indicates their self-renewal ability (data not shown). In striking contrast to the CD24^+^ cells, CD24^−^ cells formed no tumorspheres. Next we tested in vivo the tumorigenic capacity of each population. The MKR female mice serve as an ideal mouse model to study the effect of hyperinsulinemia on cancer. These mice are hyperinsulinemic with severe insulin resistance. Importantly these mice are non-obese, hence, free of the inflammatory and other responses accompanied with obesity [19].

CD24^−^ and CD24^+^ cells were implanted into the fourth mammary fat pad of WT and MKR female mice. Interval measurements of tumor volume over a period of 5 weeks were performed in addition to measurements of tumor weight at necropsy. Along with previous results, both CD24^−^ and CD24^+^ cells formed significantly larger tumors in the MKR mice compared to WT mice (2.25-fold and 1.6-fold larger, respectively) (Fig. 2c). Moreover, CD24^+^ cell tumors had an enhanced tumor growth rate compared to the CD24^−^ cell tumors in both WT (3.8-fold) and MKR mice (2.75-fold) (Fig. 2c). Terminal tumor weights confirmed these results; both CD24^−^ and CD24^+^ cells formed significantly larger tumors in the MKR mice (2.6-fold and 1.7-fold, respectively). In addition, in both WT and MKR mice CD24^+^ formed significantly larger tumors compared to the CD24^−^ cells (3.99-fold and 2.66-fold fold, respectively) (Fig. 2d).

### Low levels of CD24 are sufficient to maintain tumorigenicity properties

**Fig. 3:**
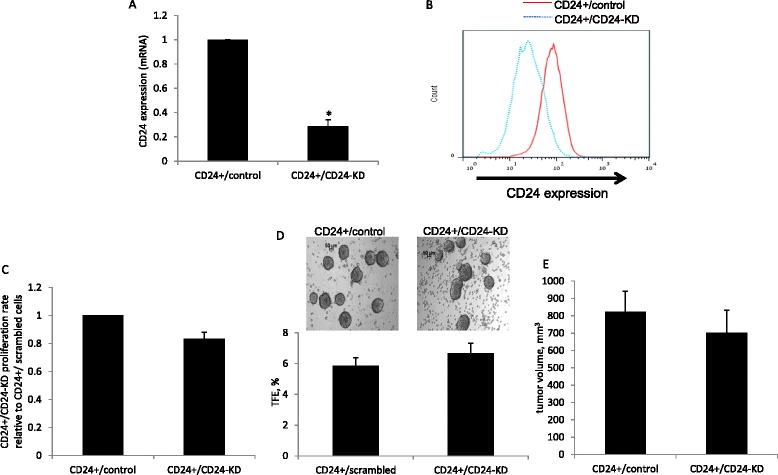
Low levels of CD24 are sufficient to maintain tumorigenicity properties. **a** Quantitative RT**-**PCR analysis of CD24 mRNA in cells infected with control or CD24 shRNA as indicated. **b** Fluorescence-activated cell sorting analysis of CD24 expression in cells transfected with control or CD24 shRNA. **c** 1*10^3^ CD24^+^/control and CD24^+^/CD24-KD cells were seeded and grown in full medium for 3 days; the relative proliferation rate between the cells was determined using the Cyquant proliferation assay. **d** Representative photomicrographs of a tumorspheres (>50 um diameter) grown from CD24^+^/control and CD24^+^/CD24-KD single cells in non-adherent culture for 5 days (*upper panel*). Percentage tumorsphere formation efficiency (*TFE %*) comparison between CD24^−^ and CD24^+^ (lower panel). **e** Cells were injected into the fourth mammary fat pad of 8-week-old virgin wild-type mice. Tumor volume was measured at necropsy. The Mann-Whitney test was performed to compare CD24^+^/control and CD24^+^/CD24-KD cells (*p <0.05)

To investigate whether CD24 serves only as a marker for tumorigenicity or is functionally involved in the induction of mitogenic pathways, we knocked down CD24 in the CD24^+^ subpopulation. The CD24 mRNA levels were significantly reduced following induction with the corresponding shRNA (Fig. 3a) and FACS analysis demonstrated a reduction in CD24 expression at the protein level (Fig. 3b). CD24 knockdown did not affect the proliferation rate of the cells, (a non-significant reduction of 17 % in the CD24^+^/CD24-KD cells compared to the CD24^+^/control cells) (Fig. 3c) nor the TFE percentage (5.85 vs 6.67 for the CD24^+^/control and CD24^+^/CD24-KD, respectively) (Fig. 3d). Although CD24 knockdown had no in vitro effect on the cells, we next determined its role in mammary tumor growth in vivo. CD24^+^/control cells and CD24^+^/CD24-KD cells were injected into the fourth mammary fat pad of WT mice after a period of 5 weeks. Both groups formed similar size tumors (824 mm^3^ and 701 mm^3^, respectively) (Fig. 3e).

### CD24^−^ cells construct the bulk of the tumors cells originating from both CD24^−^ and CD24^+^ cells

**Fig. 4:**
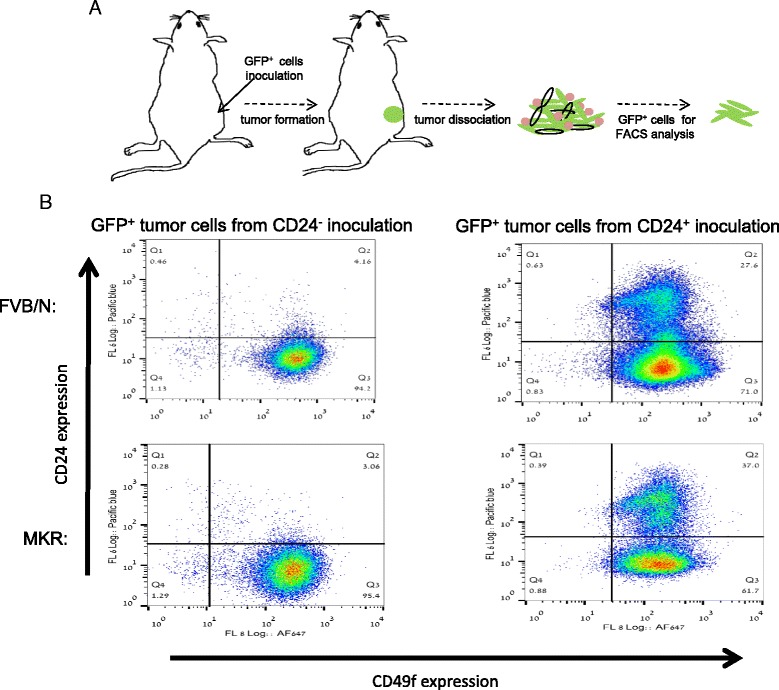
CD24^−^ cells construct the bulk of the tumors cells originating from both CD24^−^ and CD24^+^ cells. **a** Experimental strategy scheme. Green fluorescent protein (*GFP*)^+^ CD24^−^ or CD24^+^ cells were injected into the mammary fat pads. Tumors were harvested and CD24 expression was evaluated in GFP^+^ cancer cells. **b** CD24 and CD49f expression in GFP^+^ cancer cells from mammary tumors was determined by fluorescence-activated cell sorting (*FACS*) analysis

To further explore CD24^+^ stem/progenitor-cell properties, we injected CD24^+^/GFP^+^ and CD24^−^/GFP^+^ cells into the mammary fat pad of WT and MKR female mice. After 5 weeks, tumors were harvested and dissociated into single-cell suspensions and GFP^+^ cells were analyzed by FACS for CD24 and CD49f expression (Fig. 4a). The CD24/CD49f profile of CD24^−^/GFP^+^ resulting tumors was nearly identical to that of the parental inoculated cells; nearly 100 % of the analyzed GFP^+^ cells remained CD24^−^/CD49f^+^ (Fig. 4b) in both WT and MKR mice. However, the CD24/CD49f profile of GFP^+^ cells dissociated from CD24^+^/GFP tumors showed a drastic shift in CD24 expression. The CD24^+^/GFP^+^ inoculated cells gave rise to CD24^−^ cells in WT mice (70 %) and MKR mice (approximately 62 %), whereas less than 40 % remained CD24^+^ in both groups of mice. Both CD24^−^ and CD24^+^ cells remained CD49f^+^ (Fig. 4b).

### CD24^+^ cells differentiate in vivo into CD24^−^ cells

**Fig. 5:**
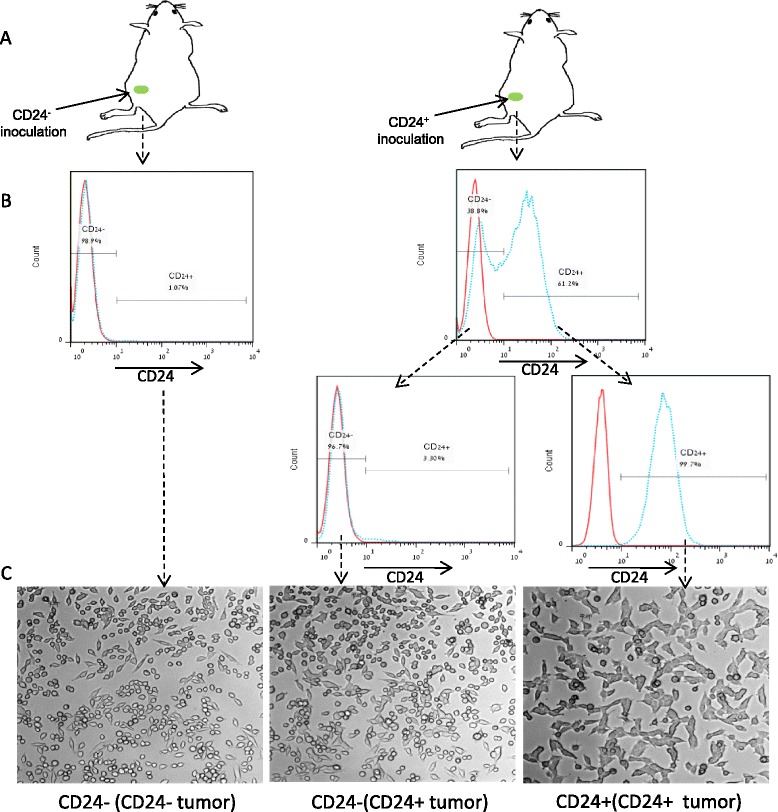
CD24^+^ cells differentiate and give rise to CD24^−^ cells in vivo. **a** Experimental strategy scheme. CD24^−^ or CD24^+^ cells were injected into the mammary fat pads; after 3 weeks, tumors were harvested and CD24 expression was evaluated. **b** CD24 expression in cancer cells from mammary tumors was determined by fluorescence-activated cell sorting analysis (*upper panel*), and CD24^−^ and CD24^+^ from CD24^+^ inoculated tumors were sorted into a pure population (*lower panel*). **c** Different phenotypes for each group shown on phase-contrast bright field images of cells grown in adherent culture

In our breast cancer model, mammary tumors became palpable within 14–21 days. To provide solid evidence for the differentiation process observed in Fig. 4b and to determine whether the abundance of CD24^−^ cells following the injection of CD24^+^ cells is an early event in tumor progression, CD24^−^ and CD24^+^ cells were injected into female mice to form mammary tumors (Fig. 5a). After 20 days, tumors were harvested and cancer cells were analyzed by FACS as described earlier for CD24 expression. As expected, injection of CD24^−^ cells resulted in an almost pure CD24^−^ population whereas approximately 40 % of the cancer cells found in the CD24^+^ inoculated tumors were CD24^−^ cells (Fig. 5b, upper panel). Next we tested whether CD24^+^ cells reduce CD24 expression and maintain tumorigenic phenotype as CD24^−^ cells or indeed differentiate into the distinct CD24^−^ subpopulation. CD24^−^ and CD24^+^ cells that were extracted from CD24^+^ inoculated tumors were sorted and subjected to cell culture growth along with CD24^−^ cells that were extracted from the CD24^−^ inoculated tumors (Fig. 5b, lower panel) and were characterized for morphology. Grown in adherent culture, both CD24^−^ subpopulations displayed a similar phenotype with flatter, more rounded epithelial cells, whereas the CD24^+^ cells had distinct mesenchymal-like morphology with more elongated cytoplasm (Fig. 5c). The in vitro phenotype of the cells was tested and as expected, extracted CD24^+^ cells had both an enhanced proliferation rate and tumorsphere forming efficiency compared to CD24^−^ cells that were extracted from both CD24^−^ and CD24^+^ tumors (Additional file 1: Figure S1).

### Gene expression analysis reveals extracellular matrix gene expression for the CD24^+^ cells

**Fig. 6:**
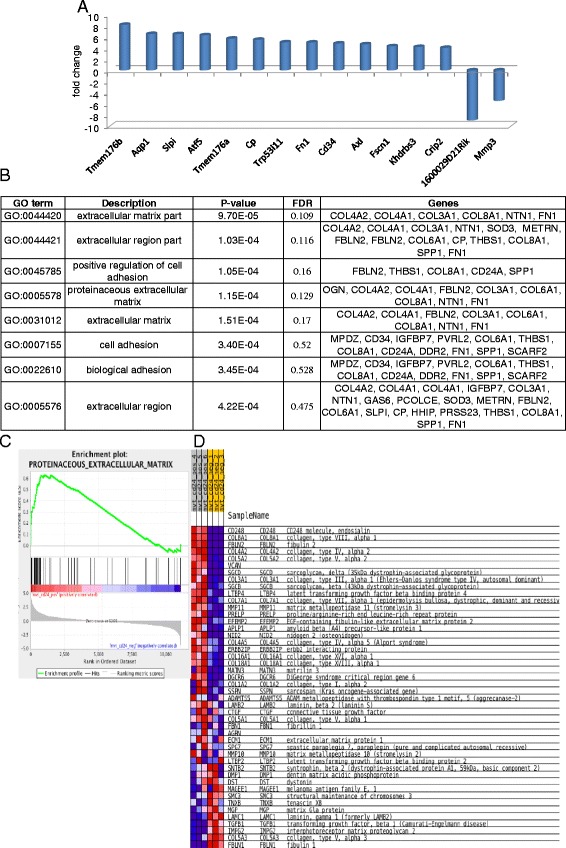
Gene expression analysis reveals extracellular gene expression for the CD24^+^ cells. **a** Top 15 alternated genes between CD24^−^ and CD24^+^ (*p* value <0.05). **b** Enriched Gene Ontology (GO) terms in CD24^+^ cells. **c** Gene set enrichment analysis display strong association between CD24 and the proteinaceous extracellular status matrix gene set. **d** Expression heat map of CD24^−^ and CD24^+^ cells. *FDR* false detection rate

In order to determine if the two Mvt-1 subpopulations (CD24^−^ and CD24^+^) gene profile support the distinct morphology of the groups and can further explain the tumorigenic capacity of the CD24^+^ cells, we analyzed the gene expression profile using RNA-seq. We identified 146 differently expressed genes (*p* value <0.05, fold-change >2) between the two groups; 89 genes were upregulated and 57 genes were downregulated in the CD24^+^ cells compared to the CD24^−^ cells (a list of the top 15 differentially expressed genes is indicated in Fig. 6a; the full list is in Additional file 2: Table S1). We found upregulation in multiple genes associated with cancer growth and invasiveness, such as AXL, a tyrosine kinase receptor that mediates tumorigenesis and metastasis; AXL was recently suggested as a potential therapeutic target in breast cancer [20, 21]. TMEM176A and TMEM176B, two transmembrane proteins that were recently found to be elevated in breast cancer and other human malignancies [22] were significantly elevated in the CD24^+^ cells. Additionally we found significantly higher levels of Twist2 in the CD24^+^ cells, a transcription factor that enhances tumor invasion by promoting an epithelial-mesenchymal transition [23]. Moreover, we found upregulation of genes that are related to invasiveness (MRC2, FSCM and SERPINH1) [24–26]. To better characterize the CD24^+^ aggressive phenotype, gene ontology (GO) enrichment analysis was applied. Metastasis formation is a multistep process, which requires detachment and embolization of cancer cells, circulation survival, extraversion and proliferation within a new organ [27]. The top GO terms enriched with a false detection rate (FDR) <0.5 in the CD24^+^ cells were associated with components of the extracellular matrix (ECM) and cell adhesion (Fig. 6b). Quantitative RT-PCR was used to independently validate the RNA-seq analysis results (Additional file 3: Figure S2). GO terms, GO:0045785 ~ positive regulation of cell adhesion and GO:0005578 ~ proteinaceous extracellular matrix (Fig. 6c) were found to be enriched in the CD24^+^ cells. These pathways were recently reported to be upregulated in distinct populations of circulating cancer cells [28]. This analysis suggests that CD24^+^ cells possess tumor-initiating properties with an advantage to thrive not only at the primary tumor site but also at a distant site by enhanced transendothelial invasion efficiency and matrix remolding at the host tissue. It is important to note that GO terms were not found for the CD24^−^ enriched genes with an FDR <5.

### CD24 cells are highly metastatic cells with enriched stem/progenitor properties in vivo

**Fig. 7:**
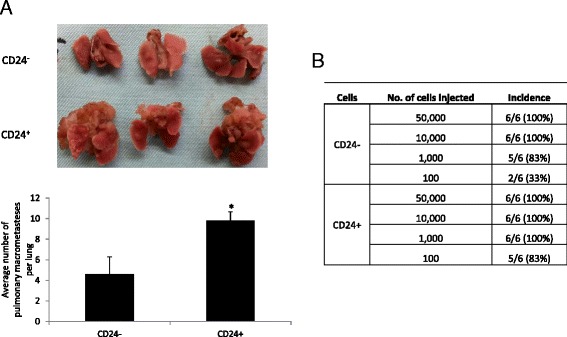
CD24 cells are highly metastatic cells with enriched stem/progenitor properties in vivo. **a** Lung metastasis after 4 weeks of inoculation of 10,000 cells into the tail vein of wild-type (WT) mice (*upper panel*); average macrometastases per lung in each group (*lower panel*). The Mann-Whitney test was performed to compare the groups (*p <0.05). **b** Cell number from the indicated population was injected into the fourth mammary fat pad of WT mice, and mammary tumor formation was monitored for a period of 5 months

To confirm CD24+ cell metastatic phenotype, we used the tail vein metastasis assay. Inoculation of each cell subset into the tail vein of WT mice revealed significantly higher metastatic capacity for the CD24^+^ cells compared to the CD24^−^ cells as indicated by the high frequency of larger lung lesions following CD24^+^ cells inoculation (Fig. 7a). To further establish the tumorigenic capacity and progenitor/stem-like features of the CD24^+^ cells, CD24^-^ or CD24^+^ cells were injected into WT mice as a dilution series (Fig. 7b). CD24^+^ cells formed mammary tumors in 83 % of the injection with as few as 100 cells, whereas CD24^−^ cells generated mammary tumors only in 33 % of the cases. These data provide clear evidence for the tumor-initiating properties of the CD24^+^ cells which enable them to generate a supported niche to proliferate in the host tissue.

## Discussion

Breast tumors comprise heterogeneous cell populations that can be characterized and isolated with specific cell surface markers [29, 30]. CD24, a known marker for mammary stem and progenitor cells, was proven useful to enrich mammary cancer stem cells [31]. CD24^+^ cells were shown to be highly tumorigenic, with capability to form mammary tumors even when implanted in low numbers [9, 32]. From a clinical perspective, CD24 was recently suggested as a prognostic marker for invasive breast tumors and has been associated with shortened disease-free survival [33, 34]. Here we studied the potential role of CD24 in the vascular epithelial growth factor (VEGF)/c-myc mutated Mvt-1 cell line. We report here that CD24 serves as a cell surface marker for highly metastatic tumorigenic cancer cells. Moreover, we demonstrated that the cancer process is orchestrated by stem/progenitor-cancer cells that account for tumor heterogeneity that is evoked by microenvironment stimuli. Using RNA-seq analysis we revealed a unique gene signature for the CD24^+^ subset, with elevated expression of extracellular matrix genes that support the highly metastatic properties of these cells. These results suggest that the extracellular matrix genes are new therapeutic targets for invasive tumors.

CD24 expression is abundantly expressed in human solid tumors; its expression is associated with high tumorigenic capacity and worse prognosis [35]. The role of CD24^+^ cells in breast tumors is unclear, whereas studies have shown that CD24^−^ cells are widely described as tumor initiating cells with stem/progenitor-like properties [15, 36]. Others have demonstrated that this CD24 phenotype is not universal for all cancer models but it is more diverse and is affected by the driver mutation [4].

In the present study, we found heterogeneity within the Mvt1 cells by characterizing the cell-surface expression of CD24; we isolated two distinct subpopulations, CD24^−^ and CD24^+^ cells. Along with other studies, CD24^+^ cells have been found to have higher proliferation rates [37], however, the underlying mechanism remain unclear. CD24 has already been suggested as a surface marker of mammary stem and progenitors cells [9]. Using the tumorsphere assay, we demonstrated that the CD24^+^ subpopulation is enriched with early stem/progenitors cells with self-renewal properties. Next, we tested the tumorigenic potential of these distinct populations in both WT and MKR mice. The MKR female mice serve as an ideal model to isolate insulin mitogenic effects; accelerated mammary gland development was found in these hyperinsulinemic female mice [17, 19]. Furthermore, orthotopic mammary tumors displayed significantly accelerated growth in these mice compared to WT mice [38, 39]. We report here that CD24^+^ cells are highly tumorigenic, forming significantly larger tumors in both WT and MKR female mice. Although CD24^+^ cells display high tumorigenic capacity, it remains to be determined whether CD24 serves as a marker for tumorigenicity or if it is functionally involved in tumorigenesis. CD24 is mostly considered as an adhesion molecule; by interacting with p-selectin. CD24 it promotes the initial steps of cell migration, and its expression in breast tumors is associated with metastasis [34, 40].

Recent studies have suggested that CD24 is involved in intracellular signaling despite the lack of an intracellular domain [41–43]. Using shRNA technology we were able to determine that in VEGF/c-myc mutated cancer cells and in the context of breast cancer, CD24 could serve as a key marker to identify a subpopulation of tumorigenic cancer cells, however it is not functionally involved in the induction of mitogenic pathways. CD24^+^/CD24-KD cells remained highly proliferative, with ability to form tumorspheres with high efficiency. Importantly, CD24-KD had no effect on tumor growth, as implantation of both CD24^+^/control and CD24^+^/CD24-KD cells formed rapidly growing tumors. It was recently demonstrated that CD24^−/−^ cells are able to functionally reconstitute cleared mammary fat pads [44]. These findings, along with our results, suggest CD24 is mainly a marker in the mammary epithelium. However, these data cannot absolutely exclude a role for CD24 in mediating the metastatic process. Whether it is the result of clonal evolution or hierarchical organization that follows the cancer stem cell model, mammary tumors along with other solid tumors constitute phenotypically and functionally heterogeneous cell populations [6, 45, 46]. With the tumorsphere assay we identified stem/progenitor activity and self-renewal properties in the CD24^+^ cells in vitro. We next tested these properties in vivo, having hypothesized that CD24^+^ cells can differentiate and give rise to CD24^−^ cells. To validate this, we implanted CD24^+^/GFP^+^ cells into the mammary fat pad of both WT and MKR mice. FACS analysis of the labeled cancer cells revealed that CD24^+^ cells fuels the cancer process by giving rise to the CD24^−^ cells that comprise the tumor bulk.

Next, we confirmed that CD24^−^ cells that were extracted from the CD24^+^ tumors were morphologically and functionally distinct from their CD24^+^ counterparts. Unlike the CD24^+^ cells, these CD24^−^ cells (that originated in vivo from the CD24^+^ cells) displayed a flatter, more rounded epithelial phenotype as oppose to the mesenchymal-like morphology of the CD24+ cells. These data are consistent with the concept of cancer stem cells having intra-tumor hierarchical organization as a result of cancer cell differentiation [47]. These findings, suggest that mammary tumors develop in a multi-step process, which is dictated by CD24^+^ cells that demonstrate directed plasticity towards the differentiated CD24^−^ cells. Moreover, the presence of about 30 % of CD24^+^ cells in the CD24^+^ inoculated tumors, suggests that the CD24^+^ cells are capable of undergoing asymmetric divisions, thus, expanding both CD24^+^ and CD24^−^ lineages. It is important to note that CD24^+^ cells were not able to differentiate in vitro even when co-cultured with tumor-derived fibroblasts (data not shown). Other tumor microenvironment factors should be evaluated in order to determine which extrinsic mechanisms promote cancer cell differentiation.

In order to identify transcripts and pathways that may elucidate the enhanced tumorigenesis upon CD24^+^ cells implantation and may serve in the future as therapeutic targets, we compared the CD24^−^ and CD24^+^ cellular transcriptome. Our study identified 157 candidates with divergent expression between these two groups. Genes that are associated with the immature state (Tmem176b, Tmem176a, ATF5) [22, 48] were significantly elevated in the CD24^+^ cells. Our analysis also identified upregulation in genes that promote proliferation (AXL and DDR2) [20, 49], migration and invasion (MRC2, FSCM and SERPINH1) [24–26], and immune response-associated genes (SLPI and CHAC1) [50, 51]. Most elevated pathways found in the CD24^+^ are involved in matrix formation and remodeling. The GO: 0005578 ~ proteinaceous extracellular matrix term was recently identified as common to distinct circulating cancer cell populations [28]. This finding suggests that CD24^+^ cells possess highly metastatic and tumor-initiating properties. We confirmed these findings in vivo with the tail vein metastasis assay and by limited dilution transplantation we provided clear evidence for the stem-like properties of the CD24^+^ cells.

## Conclusions

Taken together, our findings demonstrate that CD24 can serve alone as a marker to identify highly tumorigenic cancer cells with early stem/progenitor-cell properties. Furthermore, CD24^+^ cells are driven to differentiate in response to intra-tumor stimuli into a distinct CD24^−^ cell population, thus, creating intra-tumor heterogeneity. Moreover, we reveal here key genes as new therapeutic targets in invasive breast tumors.

## Additional files

Additional file 1: Figure S1. Ex vivo phenotype of CD24^−^ and CD24^+^ cells. Proliferation rates and percentage tumorsphere formation efficiency (TFE) of CD24^−^ cell extracted from CD24^−^ developed tumors and CD24^−^ and CD24^+^ cells extracted from CD24^+^ developed tumors were compared. **A** Cells were seeded and grown in full medium for 2 days, and the relative proliferation rates between the cells were determined using the Cyquant proliferation assay. **B** Representative photomicrographs of a tumorspheres (>50 um diameter) grown from CD24^−^ and CD24^+^ single cells in non-adherent culture for 5 days (upper panel). TFE (%) comparison between CD24^−^ and CD24^+^ (*lower panel*). Additional file 2: Table S1. Differentially expressed genes between CD24^+^ and CD24^−^ cells. Additional file 3: Figure S2. Differential gene expression for the CD24^−^ and CD24^+^ cells by qRT-PCR. RNA was extracted from independent samples of CD24^−^ and CD24^+^ cells, cDNA was synthesized, and relative mRNA expression of the indicated genes was determined using qRT-PCR.

## Abbreviations

bp: base pairs
BSA: bovine serum albumin
DMEM: Dulbecco’s modified Eagle’s medium
FACS: fluorescence-activated cell sorting
FBS: fetal bovine serum
FDR: false detection rate
GFP: green fluorescent protein
GO: Gene Ontology
PBS: phosphate-buffered saline
SEM: standard error of the mean
ShRNA: short hairpin RNA
TFE: tumorsphere formation efficiency
