# Supplementary material for: CD24+ cells fuel rapid tumor growth and display high metastatic capacity
Source: Breast Cancer Res. 2015 Jun 4;17(1):78. doi: 10.1186/s13058-015-0589-9 (PMC4479226; doi:10.1186/s13058-015-0589-9)
Supplement: Additional file 1: Figure S1. — Ex vivo phenotype of CD24− and CD24+ cells. Proliferation rates and percentage tumorsphere formation efficiency (TFE) of CD24− cell extracted from CD24− developed tumors and CD24− and CD24+ cells extracted from CD24+ developed tumors were compared. A Cells were seeded and grown in full medium for 2 days, and the relative proliferation rates between the cells were determined using the Cyquant proliferation assay. B Representative photomicrographs of a tumorspheres (>50 um diameter) grown from CD24− and CD24+ single cells in non-adherent culture for 5 days (upper panel). TFE (%) comparison between CD24− and CD24+ (lower panel). [file 13058_2015_589_MOESM1_ESM.pptx]

## Slide 1
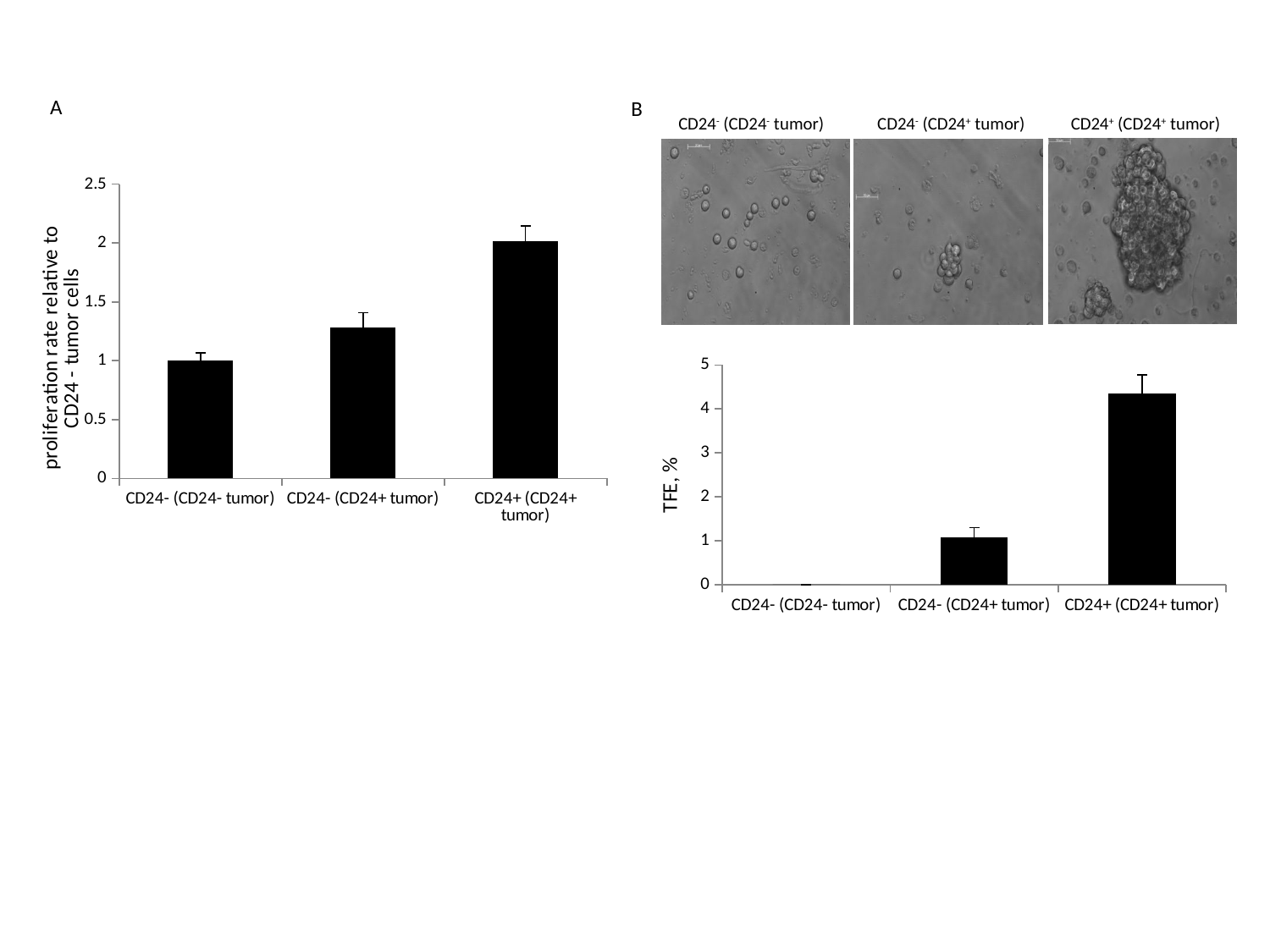

A
B
CD24- (CD24- tumor)
CD24+ (CD24+ tumor)
CD24- (CD24+ tumor)
### Chart
| Category | |
|---|---|
| CD24- (CD24- tumor) | 0.0 |
| CD24- (CD24+ tumor) | 1.0714285714285714 |
| CD24+ (CD24+ tumor) | 4.357142857142858 |
### Chart
| Category | |
|---|---|
| CD24- (CD24- tumor) | 0.9999999999535856 |
| CD24- (CD24+ tumor) | 1.2822359703919124 |
| CD24+ (CD24+ tumor) | 2.0130161253640435 |
