# Supplementary material for: CD24+ cells fuel rapid tumor growth and display high metastatic capacity
Source: Breast Cancer Res. 2015 Jun 4;17(1):78. doi: 10.1186/s13058-015-0589-9 (PMC4479226; doi:10.1186/s13058-015-0589-9)
Supplement: Additional file 2: Table S1. — Differentially expressed genes between CD24+ and CD24− cells. [file 13058_2015_589_MOESM2_ESM.docx]

Table S1: Differentially expressed genes between CD24^+^ and CD24^-^ cells.

| **Genes with elevated expression in CD24^+^ cells** | | |
| --- | --- | --- |
| **Gene symbol** | **Fold change** | **P value** |
| Tmem176b | 8.2192 | 0.017 |
| Aqp1 | 6.5614 | 0.002 |
| Slpi | 6.5387 | 0.021 |
| Atf5 | 6.3291 | 0.033 |
| Tmem176a | 5.7239 | 0.026 |
| Cp | 5.5213 | 0.022 |
| Trp53i11 | 5.0490 | 0.047 |
| Fn1 | 5.0211 | 0.014 |
| Cd34 | 4.8467 | 0.008 |
| Axl | 4.6946 | 0 |
| Fscn1 | 4.3349 | 0.001 |
| Khdrbs3 | 4.2281 | 0.028 |
| Crip2 | 4.0615 | 0 |
| Asns | 3.9041 | 0.002 |
| Mtap | 3.7685 | 0.007 |
| Col4a1 | 3.7659 | 0 |
| Trib3 | 3.7295 | 0.078 |
| Chst1 | 3.3450 | 0.001 |
| Spp1 | 3.3357 | 0.002 |
| Psmb8 | 3.2671 | 0.001 |
| Psph | 3.2603 | 0.041 |
| Trim35 | 3.1318 | 0.002 |
| Ddit4 | 3.1275 | 0.001 |
| Chac1 | 3.0801 | 0.031 |
| Sod3 | 3.0801 | 0 |
| Mrc2 | 3.0759 | 0.007 |
| Sdpr | 3.0441 | 0.002 |
| Ntn1 | 2.9160 | 0.006 |
| Mthfd2 | 2.8959 | 0.003 |
| Col6a1 | 2.8639 | 0.029 |
| Serpinh1 | 2.8540 | 0.009 |
| Atf4 | 2.8402 | 0.004 |
| Ddr2 | 2.7934 | 0.003 |
| Pcolce | 2.7934 | 0.002 |
| Fam176b | 2.7664 | 0.013 |
| Col8a1 | 2.7587 | 0.007 |
| Tceal8 | 2.7170 | 0.005 |
| Fads2 | 2.6299 | 0.009 |
| Aldh1l2 | 2.6299 | 0.018 |
| Igfbp7 | 2.6190 | 0.025 |
| Gas6 | 2.5704 | 0.021 |
| Col4a2 | 2.5669 | 0 |
| Pck2 | 2.5527 | 0.014 |
| Slc3a2 | 2.4880 | 0.016 |
| Spry1 | 2.4640 | 0.04 |
| Gpt2 | 2.4589 | 0.013 |
| Colec12 | 2.4470 | 0.008 |
| Pvrl2 | 2.4267 | 0.024 |
| Fads1 | 2.4233 | 0.014 |
| Aspa | 2.4183 | 0.034 |
| Slc7a5 | 2.3966 | 0.026 |
| Sdc2 | 2.3883 | 0.004 |
| Cd24a | 2.3603 | 0.018 |
| Cbr2 | 2.3506 | 0.046 |
| Cotl1 | 2.3359 | 0 |
| Cnn2 | 2.3311 | 0.013 |
| Aars | 2.3134 | 0.01 |
| Mgst1 | 2.3070 | 0.018 |
| Col3a1 | 2.2768 | 0.04 |
| Ppic | 2.2752 | 0.007 |
| Hhip | 2.2548 | 0.008 |
| Prss23 | 2.2548 | 0.008 |
| Mpdz | 2.2130 | 0 |
| Fabp5 | 2.1886 | 0 |
| Fah | 2.1510 | 0.036 |
| Mgat3 | 2.1495 | 0.005 |
| Nr2f1 | 2.1480 | 0.005 |
| Sh3d19 | 2.1332 | 0.002 |
| Adh7 | 2.1302 | 0.001 |
| Lmo4 | 2.1082 | 0 |
| Tars | 2.1067 | 0.003 |
| Foxf1a | 2.0980 | 0.008 |
| Nupr1 | 2.0835 | 0.042 |
| Nqo1 | 2.0835 | 0.024 |
| Rgl1 | 2.0720 | 0.002 |
| Hspa1a | 2.0691 | 0.005 |
| Twist2 | 2.0648 | 0.013 |
| 2700007P21Rik | 2.0548 | 0.02 |
| Scarf2 | 2.0463 | 0 |
| Nrn1 | 2.0293 | 0.017 |
| Csrp1 | 2.0251 | 0.039 |
| Ptprn | 2.0153 | 0.025 |
| Metrn | 2.0139 | 0.008 |
| Fbln2 | 2.0111 | 0.001 |
| Trim44 | 2.0083 | 0.01 |
| Fkbp10 | 2.0069 | 0.018 |
| Lhfp | 1.9958 | 0.018 |
| Mme | 1.9931 | 0 |
| Syt13 | 1.9917 | 0.007 |
| **Genes with reduced expression in CD24^+^ cells** | | |
| 1600029D21Rik | 8.9818 | 0.051 |
| Mmp3 | 5.4679 | 0.004 |
| Serpinb9b | 3.8557 | 0.041 |
| Selp | 3.7218 | 0.012 |
| Myc | 3.4462 | 0.002 |
| Krt18 | 3.3893 | 0 |
| Ctsl | 3.3589 | 0.001 |
| Perp | 3.3543 | 0.003 |
| Padi4 | 3.3196 | 0.038 |
| Lat2 | 3.2875 | 0 |
| Hmha1 | 3.1689 | 0.031 |
| Cd68 | 3.1167 | 0.047 |
| Mt1 | 3.0251 | 0.027 |
| Anxa8 | 2.9752 | 0.03 |
| Mt2 | 2.9588 | 0.073 |
| Rnf128 | 2.9485 | 0.003 |
| Fndc3c1 | 2.8859 | 0.016 |
| Afap1l1 | 2.8343 | 0.003 |
| Dusp2 | 2.7914 | 0 |
| Lamb3 | 2.7038 | 0.043 |
| Aebp1 | 2.6703 | 0.043 |
| Podxl | 2.5385 | 0.001 |
| Ano1 | 2.4217 | 0.001 |
| Itga6 | 2.4099 | 0.024 |
| Aim2 | 2.3751 | 0.003 |
| Serpinb6a | 2.3603 | 0 |
| Eef1e1 | 2.3424 | 0.02 |
| Rnpep | 2.3408 | 0.008 |
| Capn2 | 2.3150 | 0.005 |
| Crip1 | 2.3134 | 0.005 |
| Atg9b | 2.3006 | 0.021 |
| Hsd3b7 | 2.2626 | 0.02 |
| Itgb7 | 2.2470 | 0.003 |
| Tagln2 | 2.2099 | 0 |
| Ucp2 | 2.1886 | 0.016 |
| Ydjc | 2.1228 | 0.011 |
| Ier3 | 2.1214 | 0.001 |
| Serinc2 | 2.1126 | 0.002 |
| Rtkn | 2.0936 | 0.004 |
| Abcg2 | 2.0734 | 0.008 |
| Wdr45 | 2.0705 | 0 |
| Krt8 | 2.0634 | 0.002 |
| Morc4 | 2.0548 | 0.025 |
| Gtpbp4 | 2.0463 | 0.022 |
| Rhob | 2.0449 | 0.029 |
| Bat2d | 2.0350 | 0.004 |
| Lipg | 2.0125 | 0.034 |
| Tgfa | 2.0097 | 0 |
| Grap | 2.0042 | 0.006 |
| 4930455F23Rik | 1.9945 | 0.001 |
| Txnip | 1.9889 | 0.064 |
| Klra33 | 1.9876 | 0.026 |
| Gpr97 | 1.9821 | 0.022 |
| Igf2bp1 | 1.9807 | 0.009 |
| Degs1 | 1.9684 | 0.003 |
| Prickle3 | 1.9670 | 0.001 |
| Uck2 | 1.9629 | 0.002 |
