# Supplementary figures and images for: CD24+ cells fuel rapid tumor growth and display high metastatic capacity
Source: Breast Cancer Res. 2015 Jun 4;17(1):78. doi: 10.1186/s13058-015-0589-9 (PMC4479226; doi:10.1186/s13058-015-0589-9)

## Slide 1
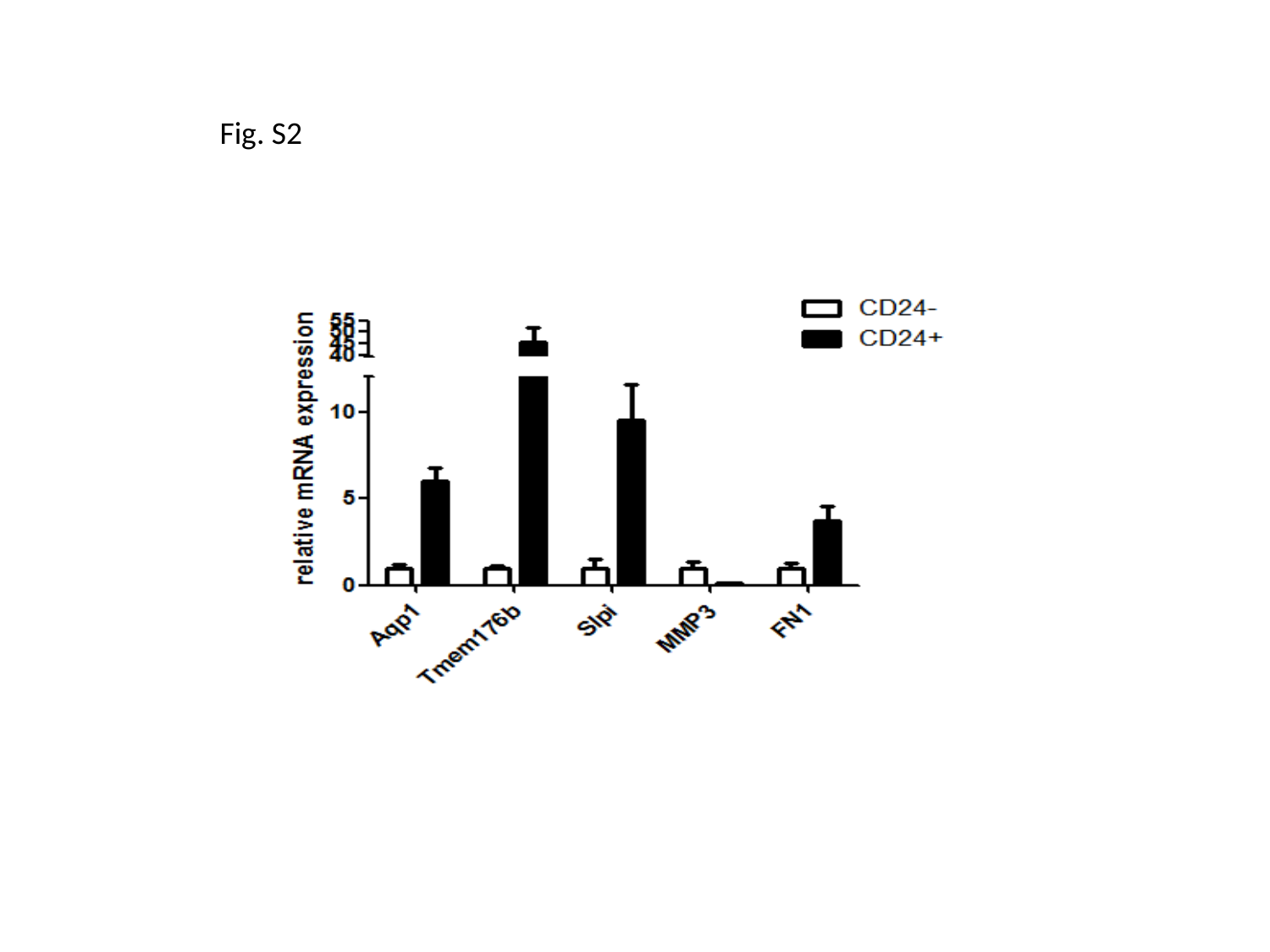

Fig. S2

Supplement: Additional file 3: Figure S2. — Differential gene expression for the CD24− and CD24+ cells by qRT-PCR. RNA was extracted from independent samples of CD24− and CD24+ cells, cDNA was synthesized, and relative mRNA expression of the indicated genes was determined using qRT-PCR. [file 13058_2015_589_MOESM3_ESM.pptx]
